# Gay, bisexual, and other men who have sex with men accessing STI clinics: Optimizing HIV PrEP implementation

**DOI:** 10.1371/journal.pone.0261705

**Published:** 2022-01-27

**Authors:** Hasina Samji, Jia Hu, Michael Otterstatter, Mark Hull, Troy Grennan, David Moore, Mark Gilbert, Rob Higgins, Jason Wong

**Affiliations:** 1 British Columbia Centre for Disease Control, Vancouver, British Columbia, Canada; 2 Faculty of Health Sciences, Simon Fraser University, Burnaby, British Columbia, Canada; 3 Public Health and Preventive Medicine, University of Toronto, Toronto, Ontario, Canada; 4 School of Population and Public Health, University of British Columbia, Vancouver, British Columbia, Canada; 5 British Columbia Centre for Excellence in HIV/AIDS, Vancouver, British Columbia, Canada; 6 Faculty of Medicine, University of British Columbia, Vancouver, British Columbia, Canada; 7 School of Public Health and Social Policy, University of Victoria, Victoria, British Columbia, Canada; SUNY Downstate Health Sciences University, UNITED STATES

## Abstract

**Background:**

Gay, bisexual and other men who have sex with men (gbMSM) who attend STI clinics represent an easily accessible population for promoting HIV prevention interventions. We examined characteristics of gbMSM STI clinic attendees to identify those who could most benefit from pre-exposure prophylaxis (PrEP).

**Setting:**

GbMSM STI clinic attendees in British Columbia (BC), Canada

**Methods:**

A clinical electronic charting system of STI clinics in BC was used to identify gbMSM from 2004 to 2017. Incident HIV cases were defined as testers who had at least one HIV-negative test and a subsequent HIV-positive test. Seroconversion rates were calculated by risk factor variables and by year. Cox proportional hazards regression was used to identify independent predictors of HIV seroconversion.

**Results:**

There were 9,038 gbMSM included, of whom 257 HIV seroconverted over the study period and 8,781 remained negative HIV testers, contributing 650.8 and 29,591.0 person-years to the analysis, respectively. The overall rate of seroconversion was 0.85 per 100 person-years (95% CI: 0.75–0.96). Incidence rates were higher among patients reporting >5 partners in the previous six months, inconsistent condom use, or having a partner living with HIV and who had a previous or concurrent diagnosis of rectal gonorrhea or rectal chlamydia. gbMSM presenting with two STIs such as rectal gonorrhea and syphilis (3.59/100 person-years [95%CI: 2.33–5.22]) or rectal chlamydia and syphilis (3.01/100 person-years [95%CI: 2.00–4.29]) had the highest incidence rates.

**Conclusion:**

gbMSM with preceding or concurrent rectal STI diagnoses or syphilis had higher rates of HIV seroconversion. The data support the inclusion of specific STI diagnoses as an indication for PrEP.

## Introduction

Gay, bisexual, and other men who have sex with men (gbMSM) are disproportionately impacted by HIV in Canada [1, 2], accounting for 50% of new HIV diagnoses in British Columbia (BC), Canada in 2019 [3]. While the absolute number of new diagnoses of HIV among gbMSM has been declining in BC, the proportion of new diagnoses attributable gbMSM has increased [4]. As such, gbMSM remain a priority group for interventions aimed at reducing HIV seroconversion, including HIV pre-exposure prophylaxis (PrEP) with emtricitabine/tenofovir disoproxil fumarate (FTC/TDF), a biomedical prevention measure which has emerged as the standard of care for HIV prevention in specific groups of gbMSM. FTC/TDF for HIV PrEP has been publicly funded in BC since January 2018. It is critical for healthcare systems to identify and prioritize gbMSM at the highest risk of acquiring HIV to maximize the impact of PrEP.

Objective and subjective markers of risk have been explored to determine parameters for PrEP eligibility among gbMSM; the HIV Incidence Risk Index for Men who have Sex with Men (HIRI-MSM) is one such tool that has been validated and used extensively in clinical practice and which has been included in numerous local and national guidelines for establishing PrEP eligibility [5]. However, existing tools and guidelines may miss or not appropriately emphasize risk factors such as recent sexually transmitted infection (STI) diagnosis and the repeated use of HIV postexposure prophylaxis, clear clinical markers that may predict future HIV acquisition and can signal clinicians and health systems to intervene [6–8]. Moreover, many tools rely on the ability of healthcare providers to elicit sensitive information, which may limit their application in settings where providers do not have experience or expertise in working with gbMSM clients. As such, combining risk factor approaches for PrEP eligibility with existing points of contact for individuals at increased risk of HIV seroconversion with the health system (e.g., gbMSM receiving an STI diagnosis) may optimize PrEP delivery [6, 7]. This work builds on these findings through a retrospective analysis of electronic health records from gbMSM in the province of British Columbia (BC), Canada.

STI diagnosis data from repeat testers has been shown to be an efficient means of identifying sub-populations most at risk for HIV and other STIs [1, 9–12]. For example, using an STI clinic sample in New York City, Pathela et al. identified race and condom use as well as incident STIs as highly associated with HIV acquisition; yet, evidence pertaining to the risk of seroconversion in the presence of multiple STIs remains limited [12]. A more nuanced role of risk associated with multiple STIs and HIV acquisition can be used to best target HIV prevention efforts. We examined characteristics of STI clinic attendees to identify gbMSM who could most benefit from PrEP in BC. As STI clinics in BC routinely collect testing and behavioural data, it is both cost-effective and efficient to derive estimates of HIV incidence from these data and to use them to identify groups at highest risk of HIV seroconversion and who therefore may benefit most from PrEP. Moreover, STI clinics may be a main point of contact with the health care system for many gbMSM. Lastly, the charting system used by these clinics also served as a comprehensive repository for selected STI diagnoses (e.g., HIV, syphilis) in the province until 2017 (the end point of our analysis) allowing for data from other sources to be included. We examined characteristics of gbMSM whose data is stored in this repository in BC to identify those who could most benefit from PrEP and to evaluate whether our findings corresponded to the Canadian guidelines establishing PrEP eligibility.

## Materials and methods

### Cohort and study population

The data for our study comes from the STI Information System (STI-IS), an electronic charting system used by STI clinics and street outreach programs operated by the provincial public health system providing sexual health care to clients for screening and diagnosis of STI [13]. Many of the clinics and programs using STI-IS as their charting system were established as a result of activism from community members who fought to have public health resources dedicated to gbMSM given HIV’s disproportionate impact on this community. Especially early in BC’s HIV epidemic, gbMSM experienced a great deal of stigma and homophobia and needed access to testing opportunities that were able to engage with them in a safe and respectful way to improve access to services. Therefore, these clinics are a highly frequented venue for gbMSM seeking testing. These clinics and outreach teams are staffed with nurses and physicians who provide counselling, testing and treatment for STIs, including HIV, in a free and confidential setting. STI-IS is also the database for documentation of all reportable STIs, including chlamydia, gonorrhea, and syphilis, diagnosed in BC. In addition to testing, behavioural risk factor data collection is undertaken by trained nurses and inputted into the STI-IS system at the time of the clinic visit. We included 39 sites distributed across the province’s five regional health authorities. We included patients who were seen between January 1, 2004 and December 31, 2017. We limited our analysis to gbMSM, defined as male patients whose sexual orientation was self-reported as homosexual or bisexual at the time of testing. Patients were included if they had at least two HIV tests, of which the first was negative. Patient visits to a clinic separated by seven days or less were assumed to be follow-up visits and therefore collapsed into a single visit.

### Outcome definition

Incident HIV cases were defined by a documented negative HIV test followed by a positive HIV test, during the study period. In calculating the person-years contributed to the time-at-risk, we used the time between each patient’s first negative test and either their last negative test or their estimated date of seroconversion. Given that we cannot know when seroconversion actually occurred, we fixed seroconversion date as the midpoint between the last negative and first positive test, which follows methodology used previously to estimate HIV seroconversion date [1, 13–15].

### Explanatory variables

Variables extracted from STI-IS included date of visit, clinic location, age, self-reported condom use pattern, partner HIV status, and number of partners in the previous six months. HIV test information and results and information on reportable STI tests and results (i.e. rectal gonorrhea, rectal chlamydia or syphilis) were also derived from a linkage to the BCCDC Public Health Laboratory. As this was an individual-level analysis and not test-level, we used the result at the time of the final HIV test (last negative or first positive test) for behavioural variables that potentially vary over time (condom use, partner HIV status, number of partners in prior six months) as the most relevant information for this analysis.

For analysis purposes, we categorized variables as follows: condom usage, past six months: no, inconsistent, consistent, or unknown; number of sexual partners, past six months: less than five partners, five to 14, more than 14, or unknown; HIV status of partners: positive, negative, or unknown; previous (within study period) or concurrent diagnosis (within 7 days of the HIV test) of syphilis, rectal chlamydia, or rectal gonorrhea: yes or no; and age group: 15 to 19, 20 to 24, 25 to 29, 30 to 39, 40 to 59, 60 years and greater (age categories match provincial surveillance reporting groups).

### Statistical analysis

We present crude incidence rates for the entire study period as well as by year. In calculating annual incidence of HIV, we assigned each seroconversion proportionally to the years within the seroconversion interval, and similarly assigned person-years of follow-up proportionally to the years within an individual’s total period of follow-up. Thus, a case whose seroconversion window is between July 1, 2012 and June 30, 2013 would contribute 0.5 cases to 2012 and 0.5 cases to 2013. For the purpose of understanding annual trends, this proportional assignment approach is preferable to arbitrary assignment of seroconversions to a single year, especially given that many (roughly 40%) seroconversion intervals spanned more than two years, and some spanned up to eight years. Confidence intervals for annual incidence were calculated using the exact method based on Poisson rates. Across the entire study period, we present unadjusted incidence rates by risk factor variable based on binomial regression models with a complementary log-log link, log-transformed person-years as an offset, and confidence intervals calculated using profile-likelihood method [16]. We also divided the study period in two periods (2004–2010 and 2011–2017) to determine whether there were temporal trends associated with the rate of HIV seroconversion over the study period. Specifically, dedicated funding for provincial expansion for support for HIV testing, engagement in HIV care and combination antiretroviral therapy (cART) began in 2010 and may have influenced testing and diagnosis of HIV [17].

Our primary analysis used Cox proportional hazards regression models treating seroconversion as the dependent variable and our behavioural risk factor variables, previous or concurrent STI (syphilis, rectal gonorrhea, or chlamydia) diagnosis, age group, year and testing rate (HIV tests per year of follow-up, log-transformed) as explanatory variables. Individuals who did not seroconvert during our study were censored at their last test date in this analysis. We used forward and backward stepwise elimination with significance levels of P = 0.4 and P = 0.1 for variables to enter and remain in the model, respectively. Model fit and the proportional hazards assumption were assessed using deviance residuals and cumulative sums of martingale residuals [18]. We present unadjusted and adjusted hazard ratios (HR) with 95% confidence intervals for each risk factor, where adjusted values are based on the final model from our stepwise elimination. Additional models were created to examine interactive effects of various STIs. For comparison, we also conducted stepwise elimination of the same explanatory variables using binomial regression models with a complementary log-log link and log-transformed person-years as an offset [19]. Statistical analyses were performed using SAS 9.4 (Cary, NC) [16].

### Ethical approval

The Clinical Research Ethics Board at the University of British Columbia reviewed and approved the study (H15-01487). This study was a retrospective analysis of medical records. Data were fully anonymized before analysis and informed consent requirements were waived as data were collected as part of routine public health and clinical services. Minors were also included in this study. Consent was not sought from their parents or guardians and this requirement was waived as the study was an anonymous retrospective analysis of health records.

## Results

Summary statistics for our study cohort by seroconversion status of 9,038 gbMSM included in our study are presented in Table 1. The median age was 33 years (interquartile range [IQR]: 27–42) and patients had a median number of three HIV tests (IQR: 2–6). In total, 39 clinics were represented in the dataset, though the majority (90%) of visits occurred at three clinics which were in the Greater Vancouver area. Over the study period, there were 257 HIV seroconversions and 8,781 remained negative HIV testers, contributing 650.8 and 29,591.0 person-years to the analysis, respectively.

**Table 1:** Summary statistics describing the study cohort, comparing HIV seroconverters to negative HIV testers, 2004 to 2017.

Variable	Seroconverters N = 257*	Negative testers N = 8781*	P-value**
**Person-years**	650.8	29591.0	
**Median age (25**^**th**^ **-75**^**th**^ **percentile)**	34 (27–41)	32 (27–42)	0.636
**Age, % (n)**			0.229
15–19 years	2.0%	1.2%	
20–24 years	11.3%	13.0%	
25–29 years	19.8%	23.4%	
30–39 years	36.2%	32.2%	
40–59 years	28.4%	26.1%	
60+ years	2.3%	4.2%	
**Median HIV tests (25**^**th**^ **-75**^**th**^ **percentile)**	4 (2–7)	3 (2–6)	0.137
**% having syphilis diagnosis (n)**	20.6%	6.7%	<0.001
**% having rectal chlamydia diagnosis (n)**	24.5%	10.8%	<0.001
**% having rectal gonorrhea diagnosis (n)**	23.4%	8.2%	<0.001
% reporting not using condoms, last 6 months (n*)	10.9% (N = 165)	16.3% (N = 5304)	0.051
% reporting 15 or more partners, last 6 months (n*)	6.7% (N = 119)	4.9% (N = 4356)	0.393
% reporting HIV positive partner, last 6 months (n*)	31.6% (N = 57)	10.9% (N = 2007)	<0.001

*note: sample sizes for self-reported risk factors are less than the total N due to non-responses; actual number noted in appropriate row

** P-values are from likelihood ratio chi-square tests (proportions) or Wilcoxon tests (medians).

**Fig 1:**
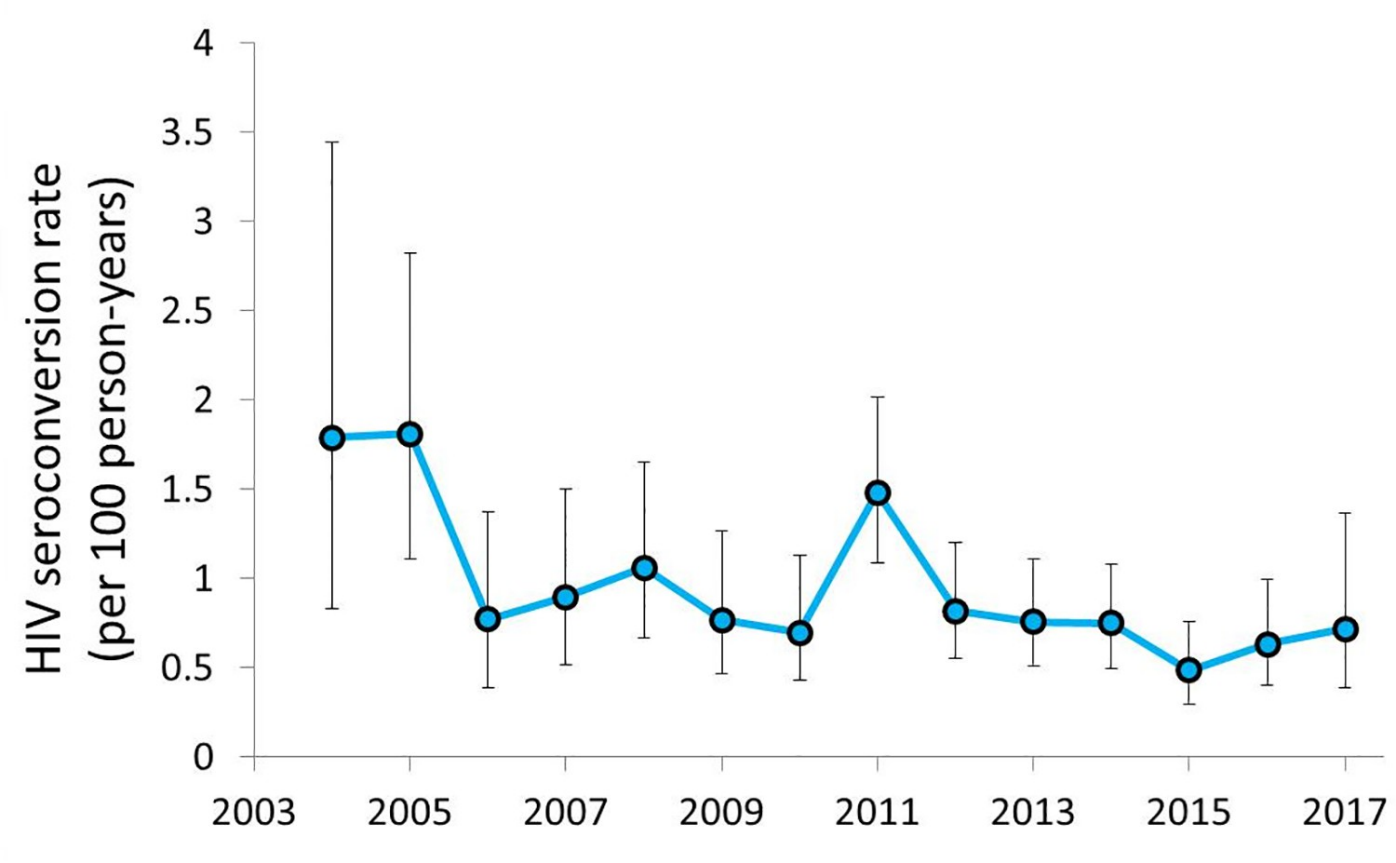
Annual HIV seroconversion rates among gay and bisexual men who have sex with men in British Columbia, 2003–2017.

The overall rate of seroconversion was 0.85 per 100 person-years (95% CI: 0.75–0.96). Seroconverters were more likely to have an STI diagnosis and an HIV-positive partner. Annual seroconversion rates are shown in Fig 1; there was a trend towards lower rates over the duration of the study period 2004–2017 (p<0.001), driven primarily by higher rates during the first two years (2004 and 2005). Rates over the remainder of the study period were relatively flat; similarly, the proportion of new diagnoses in the dataset that were gbMSM was consistently high (mean ± SD percent gbMSM: 88.5 ± 11.3%).

Dividing the study period in equal halves, which also corresponds to the launch of an important provincial HIV treatment and prevention initiative in 2010 [17], 97 seroconversions occurred during the early (2004–2010) period and 160 during the late (2011–2017) period, corresponding to rates of 0.96 (95% CI: 0.79–1.17) and 0.79 (95% CI: 0.68–0.92) seroconversions per 100 person-years respectively. Testers in the latter period were younger and had a greater median number of tests. Further descriptive statistics for the early and late periods are shown in Table 2.

**Table 2:** Descriptive statistics comparing the early (2004 to 2010) and late (2011 to 2017) halves of the study period.

Variable	Early period (2004–2010)	Late period (2011–2017)	P-value**
Seroconversions/person-years	0.95 per 100 person-years (97/10117)	0.80 per 100 person-years (160/20108)	0.149
Median age (25^th^ -75^th^ percentile)	34 (16–78)	32 (16–90)	0.043
Median HIV tests (range)	2 (2–22)	4 (2–56)	<0.001
% reporting not using condoms, last 6 months (n*)	15.6% (168/1075)	16.3% (715/3679)	0.606
% reporting ≥15 partners, last 6 months (n*)	5.4% (52/965)	4.8% (170/3510)	0.494
% reporting HIV positive partner, last 6 months (n*)	12.7% (37/291)	11.3% (200/1773)	0.482

* note: sample sizes for self-reported risk factors are less than the total n due to non-responses

** P-values are from chi-square tests (proportions) or Wilcoxon tests (medians).

Unadjusted incidence rates per 100 person-years are presented in Table 3. Rates were higher for patients: 15–19 years of age (IR: 5.26, 95% CI: 1.92–10.96); with 5 to 14 (IR: 1.33, 95% CI: 0.94–1.81) or ≥15 partners (IR: 1.19, 95% CI: 0.55–2.20) in the previous six months; reporting inconsistent condom use (IR: 1.08, 95% CI: 0.86–1.33); reporting an HIV-positive partner (IR: 1.90, 95% CI: 1.15–2.90), and who ever had a diagnosis of rectal gonorrhea (IR: 1.93, 95% CI: 1.49–2.46), syphilis (IR: 1.91, 95% CI: 1.45–2.47) or rectal chlamydia (IR: 1.56, 95% CI: 1.21–1.97). Patients presenting with two STIs had higher incidence rates; specifically, rectal gonorrhea and syphilis diagnoses (IR: 3.59, 95% CI: 2.33–5.22), rectal chlamydia and syphilis diagnoses (IR: 3.01, 95% CI: 2.00–4.29), and rectal gonorrhea diagnosis and rectal chlamydia (IR: 2.43, 95% CI: 1.70–3.34). Lowest rates were observed for patients reporting no condom use, those with an HIV-negative partner, those aged 60 years and older, and those without a previous STI diagnosis.

**Table 3:** Unadjusted incidence rates for HIV seroconversion, by risk factor, during the study period 2004–2017 (N = 9,038 individuals).

Risk factor	Incidence rate per 100 person-years (95% CI)
**Condom use**	
Consistent	0.87 (0.68–1.09)
No	0.54 (0.33–0.83)
Inconsistent	1.08 (0.86–1.33)
Unknown	0.79 (0.64–0.97)
**Sexual partners**	
< 5	0.70 (0.55–0.87)
5 to 14	1.33 (0.94–1.81)
15 or more	1.19 (0.55–2.20)
**Previous or concurrent STI**	
None	0.65 (0.55–0.76)
Syphilis	1.91 (1.45–2.47)
Rectal chlamydia	1.56 (1.21–1.97)
Rectal gonorrhea	1.93 (1.49–2.46)
Syphilis and rectal chlamydia	3.01 (2.00–4.29)
Syphilis and rectal gonorrhea	3.59 (2.33–5.22)
Rectal chlamydia and rectal gonorrhea	2.43 (1.70–3.34)
**Partner status**	
HIV positive	1.90 (1.15–2.90)
HIV negative	0.56 (0.40–0.75)
**Age group**	
15 to 19	5.26 (1.92–10.96)
20 to 24	1.53 (1.04–2.15)
25 to 29	0.97 (0.73–1.26)
30 to 39	0.89 (0.72–1.08)
40 to 59	0.70 (0.55–0.87)
60 and over	0.31 (0.12–0.62)

Analysis of risk factor variables using Cox proportional hazards regression found that HIV seroconversion was independently associated with having a previous or concurrent diagnosis of syphilis (adjusted HR: 1.98; 95%CI: 1.43–2.75), rectal gonorrhea (aHR: 1.83; 95%CI: 1.30–2.57) or rectal chlamydia (aHR: 1.71, 95% CI: 1.21–2.41). Reporting an HIV-positive sexual partner was also significantly associated with HIV seroconversion (aHR: 1.98; 95%CI: 1.12–3.49). In contrast, the number of recent (last 6 months) sexual partners, recent condom usage and age were no longer statistically significant after adjusting for STI diagnosis and an HIV-positive sexual partner and were excluded from the final model. Regression statistics are shown in Table 4.

**Table 4:** Unadjusted and adjusted hazard ratios of predictors of HIV seroconversion risk from 2004 to 2017 (N = 9,038).

Effect	Unadjusted HR (95% CI)	P-value	Adjusted HR (95% CI)	P-value
**Condom usage, last 6 months**				
No vs. Consistent	0.65 (0.39–1.10)	0.108	0.72 (0.43–1.23)	0.229
Inconsistent vs. Consistent	1.27 (0.92–1.76)	0.146	1.21 (0.87–1.69)	0.254
Unknown vs. Consistent	0.93 (0.68–1.27)	0.627	0.86 (0.61–1.21)	0.375
**Syphilis diagnosis**	2.66 (1.96–3.59)	<0.001	1.98 (1.43–2.75)	<0.001
**Rectal gonorrhea diagnosis**	2.66 (2.00–3.55)	<0.001	1.83 (1.30–2.57)	<0.001
**Rectal chlamydia diagnosis**	2.10 (1.58–2.79)	<0.001	1.71 (1.21–2.41)	0.003
**HIV status of partner**				
Positive vs. Negative	3.37 (1.93–5.89)	<0.001	1.98 (1.12–3.49)	0.018
Unknown vs. Negative	1.58 (1.12–2.22)	0.009	1.09 (0.76–1.57)	0.628
Number of sexual partners*				
5 to 14 vs. less than 5	1.88 (1.26–2.79)	0.002		
15+ vs. less than 5	1.71 (0.83–3.55)	0.149		
Unknown vs. less than 5	1.26 (0.95–1.67)	0.112		
Age group*				
20–24 vs 15–19	0.30 (0.12–0.78)	0.014		
25–29 vs. 15–19	0.20 (0.08–0.51)	<0.001		
30–39 vs. 15–19	0.20 (0.08–0.49)	<0.001		
40–59 vs. 15–19	0.16 (0.06–0.40)	<0.001		
60+ vs. 15–19	0.07 (0.02–0.24)	<0.001		

Variables denoted with * were not included in final model based on stepwise elimination. All models controlled for year and individual testing rate.

Additional regression analyses were conducted to explore the effect of having multiple previous or concurrent STI diagnoses on HIV seroconversion. Of the 257 individuals with HIV seroconversion, 23 (8.9%) had been diagnosed with both rectal gonorrhea and syphilis, 33 (12.8%) with rectal gonorrhea and rectal chlamydia, and 26 (10.1%) with syphilis and rectal chlamydia. Among HIV seroconverters, roughly 80% of STI diagnoses (syphilis, rectal chlamydia and rectal gonorrhea) occurred within 12 months prior to HIV diagnosis; the remaining occurred within seven days of HIV diagnosis and were considered concurrent with HIV. Risk factor results from the binomial regression models were consistent with those from our proportional hazards models and are not discussed further.

## Discussion

Our study investigated HIV seroconversion risk among gbMSM in the STI-IS database over a 14-year period to identify populations who would benefit most from PrEP. In adjusted models we found that reporting an HIV-positive partner and previous or concurrent diagnosis of rectal gonorrhea, syphilis or rectal chlamydia increased the risk of HIV seroconversion. HIV incidence was between 3–6 times higher among individuals with multiple previous or concurrent bacterial STIs compared to those without a previous STI diagnosis during the study period. In the Canadian context, preliminary findings from our work informed national and provincial eligibility guidelines for PrEP initiation to include gbMSM diagnosed with infectious syphilis or rectal bacterial STI in the past 12 months [6, 20]. There were 257 seroconversions among more than 9,000 individuals resulting in an overall HIV incidence rate of 0.85 per 100 person-years. Annual incidence rates remained largely consistent over the study period from 2004 to 2017, as did patient characteristics such as number of sexual partners, median age, and condom use, though the median number of tests increased over time.

Our findings underscore the need to prioritize gbMSM with a history of bacterial STIs for additional prevention resources, such as counselling, education, monitoring; referral for biomedical strategies such as PrEP; and inclusion in criteria being used to determine eligibility for PrEP initiation. As many STIs are reportable in BC and elsewhere, with follow-up mandated by the healthcare system, STI diagnosis represents a natural opportunity to connect with patients at risk of seroconversion and perform an assessment for PrEP eligibility. Moreover, we found that existing criteria for PrEP eligibility such as condom use and number of sexual partners were not predictive of HIV seroconversion; these subjective criteria may also be misleading for patients to assess their own risk of acquisition.

Based on these findings, objective clinical markers such as previous STI diagnoses should be prioritized and trigger conversations about PrEP referral, with STI clinics at the forefront of PrEP screening and referral. Additionally, these findings indicate that further research into altering PrEP eligibility criteria for gbMSM is warranted. Current guidelines require that gbMSM report having had condomless anal intercourse in addition to a rectal bacterial STI diagnosis to be eligible. While some prescribing physicians may be more lenient and flexible in their referral PrEP initiation, less experienced clinicians may more strictly adhere to these guidelines, potentially excluding patients who would benefit from PrEP. As well, due to historical stigma experienced by gbMSM in relation to HIV/AIDS, some clients may not be willing to disclose that they are engaging in condomless anal intercourse. Thus, an objective measure of risk–such as a recent rectal bacterial STI diagnosis–may more accurately capture the risk of HIV acquisition that a client is experiencing and better protect those who are more vulnerable. Such a change in guidelines would ideally position STI clinics as PrEP referral sites, with protocols in place prioritizing PrEP initiation among clients with bacterial STIs.

In addition, though the sample size was small, we found that younger gbMSM between the ages of 15 to 24 years had relatively high HIV incidence rates, supporting PrEP eligibility criteria prioritizing younger gbMSM. Not only are younger, minority gbMSM at higher risk of seroconversion [12, 21, 22] but screening for HIV and other STIs at STI clinics may be one of their few points of engagement with the healthcare system. Thus, STI clinics may be optimally suited to target those with a history of STIs and younger gbMSM in increased risk reduction counseling and referral for PrEP [23].

Our study identified a doubling of risk of HIV seroconversion for individuals with a previous or concurrent bacterial STI. Similarly, a case control study of gbMSM in a Seattle clinic identified a more than two-fold increase in the odds of HIV seroconversion associated with rectal gonorrhea and concurrent rectal chlamydia [24], while Desai et al.’s study in England examining acute bacterial STI diagnosis in the previous year found a four-fold higher risk of HIV seroconversion among those with syphilis and a two-fold increase related to gonorrhoea [25]. In a study based in New York City, annual HIV incidence was higher among gbMSM with rectal infections (6.7%) than gbMSM without rectal infections (2.5%), and a study of gbMSM in Atlanta study found that rectal STIs were associated with a more than two-fold risk of subsequent HIV acquisition [15].

HIV seroconversion was considerably higher in our study for gbMSM with two bacterial STIs occurring concurrently, results that are corroborated in a small study of gbMSM that found an 8-fold greater risk of seroconversion with two or more prior infections of gonorrhea or chlamydia [26]. Another American study found a 2.46 [95%CI: 2.01–3.00] times increased risk of HIV diagnosis among men with primary and secondary syphilis who also had a concurrent or subsequent bacterial STI versus those with only syphilis [12]. More work is needed using larger samples to confirm indications of increased risk associated with multiple STI co- or subsequent occurrence given physiological evidence linking bacterial STIs to increased risk of HIV seroconversion, likely through inflammation processes that increase mucosal disruption [24]. Increased understanding of biological and behavioural mechanisms underpinning transmission risk associated with multiple STI infections will also elucidate the nature and duration of the risk associated with each bacterial STI.

Heightened seroconversion risk among individuals with a bacterial STIs is especially concerning in the context of the ongoing syphilis epidemics among gbMSM in urban centres in North American and Europe [27], as well as increasing rates of gonorrhea and chlamydia [22, 28]. These increases have been explained as a return to pre-HIV high STI incidence in gbMSM populations as worries about HIV risk wane in the context of effective antiretroviral treatment and prevention fatigue [29]. The promise of PrEP in clinical and applied settings [30–33] in reducing risk of HIV acquisition, as well as increased STI testing, will likely reinforce this increasing trend of bacterial STIs among gbMSM, with potential implications for downstream increases in the broader population. As such, novel prevention tools such as chemoprophylaxis for bacterial STIs that address increases in STI concomitant with HIV incidence decrease may be warranted [34–36].

Our estimate of HIV incidence is comparable to a mathematical modeling study estimating population-level incidence among gbMSM in England and Wales from 2001 to 2007 (0.9%) [37] and lower compared to studies of gbMSM tested in STI clinics such as a study of repeat testers in a cohort of gbMSM testing in STI clinics in England (2012 to 2013), which found an HIV incidence of 2.0 per 100 person-years [25], and American (2007 to 2012) and Australian (2007 to 2013) studies estimating HIV incidence of 2.4 and 1.3 per 100 person-years, respectively [12, 38]. Findings are also in line with a cohort study of gbMSM conducted between 2012–2015 in Vancouver, BC, which reported an incidence rate of 1.1 per 100 person-years [39] but lower compared to an incidence estimate of 2.8 per 100 person-years in a cohort of gbMSM in Lisbon from 2011 to 2014 [40], 2.4 per 100 person-years in Barcelona from 2008 to 2011 [41], and a 1.6 per 100 person-years in a study that combined empirical methods in San Francisco in 2010 [42].

Ultimately, in a promising era where considerable progress has been achieved in reaching global HIV elimination goals, reducing HIV incidence among gbMSM remains a priority. It is possible that rising STI trends among gbMSM in BC are off-setting any decreases in HIV incidence due to cART [28]. As well, PrEP was not provincially publicly funded in BC until January 2018; as such, we may see greater reductions in HIV incidence as the program expands, as seen in other jurisdictions such as in New South Wales, Australia, where a 25.1% reduction in HIV diagnoses was observed in the 12 month period after PrEP roll-out [43, 44]. To maximize this potential reduction, bacterial STIs should be given stronger consideration in PrEP eligibility criteria.

There are several limitations to our study. Firstly, repeat testing data results in interval censoring in which disease onset occurs sometime between a last negative screen and HIV diagnosis; this is a known limitation of this type of data. Left censoring, when an outcome occurs prior to the study observation period, may also occur if disease was prevalent but not yet diagnosed. Those who are more frequently tested such as individuals frequenting STI clinics are likely at higher risk of HIV seroconversion than the broader community, and therefore incidence estimates may not be generalizable to the wider population [45]; as well, our study did note an increase in testing in later years. While clinics from across the province were included, the vast majority of gbMSM included in our study resided in Vancouver which again, may limit our ability to estimate HIV incidence for the province. However, approximately 70% of new HIV diagnoses reside in the Greater Vancouver area and thus, represent the majority of HIV burden in BC [4]. As well, patients with only one HIV test were not included in the analysis, leading to an underestimation of HIV incidence as seroconversions associated with these single testers were not included. Using behavioural data at the time of last test assumes that sexual risk variables are static and does not reflect possible changes in sexual trajectories.

Lastly, given the intersecting epidemics and shared sexual transmission routes of bacterial STIs and HIV, improved characterization of contextual factors not collected in this study such as unstable housing, stigma, poverty, social isolation, mental health status, interpersonal violence and substance use and their combined effects on risk of HIV acquisition may yield dividends in reducing incidence of all of these diseases. Such a syndemic approach has been instrumental in elucidating interacting factors leading to increased risk of HIV among populations of gbMSM [4, 46] and has been recently used to understand the risk of syphilis acquisition in BC [47]. While biomedical interventions such as pre- and post-exposure prophylaxis, male circumcision and most recently, long-acting injectable antiretrovirals [48] have either demonstrated efficacy or potential promise for prevention, fundamental causes underlying risk behaviours for HIV acquisition must continue to be addressed.

We found that objective measures such as previous STI infection were better at predicting risk of HIV seroconversion than self-reported measures such as condom use or number of sexual partners. Screening tools may benefit from considering these parameters in their indices and eligibility criteria. As gbMSM with a previous or concurrent STI diagnosis and those with an HIV-positive partner had a noticeably higher risk of seroconversion, they should be prioritized for interventions aimed at reducing HIV and STI incidence.
